# Box-Wilson Design for Optimization of *in vitro* Levan Production and Levan Application as Antioxidant and Antibacterial Agents

**DOI:** 10.52547/ibj.25.3.202

**Published:** 2020-12-27

**Authors:** Rukman Hertadi, Nur Umriani Permatasari, Enny Ratnaningsih

**Affiliations:** 1 Biochemistry Research Division, Faculty of Mathematics and Natural Sciences, Bandung Institute of Technology, Indonesia;; 2 Chemistry Department, Faculty of Mathematics and Natural Sciences, Hasanuddin University, Indonesia

**Keywords:** Antioxidants, Fructans, In vitro technique, Levan

## Abstract

**Background:**

Levan or fructan, a polysaccharide of fructose, is widely used in various commercial industries. Levan could be produced by many organisms, including plants and bacteria. The cloning of the gene from *Bacillus licheniformis*, which expressed levansucrase in *Escherichia coli* host, was carried out successfully. In the present study, we performed the *in vitro *production of levan and analyzed its potential application as antibacterial and antioxidant agents.

**Methods:**

*In vitro* levan production catalyzed by heterologous-expressed levansucrase Lsbl-bk1 and Lsbl-bk2 was optimized with BW design. The antibacterial activity of the produced levan was carried out using agar well diffusion method, while its antioxidant activity was tested by free radical scavenging assays.

**Results:**

The optimum conditions for levan production were observed at 36 °C and pH 7 in 12% (w/v) sucrose for levansucrase Lsbl-bk1, while the optimum catalysis of levansucrase Lsbl-bk2 was obtained at 32 ^o^C and pH 8 in the same sucrose concentration. The *in vitro* synthesized levan showed an antibacterial activity within a concentration range of 10-20% (w/v) against *Staphylococcus aureus*, *E. coli*, and *Pseudomonas aeruginosa*. The same levan was also able to inhibit the DPPH radical scavenging activity with the antioxidant strength of 75% compared to ascorbic acid inhibition.

**Conclusion:**

Our study, therefore, shows that the optimized heterologous expression of levansucrases encoded by Lsbl-bk1 and Lsbl-bk2 could open the way for industrial levan production as an antibacterial and antioxidant agent.

## INTRODUCTION

Levansucrase, an enzyme belonging to the fructosyltransferase class, is secreted by several organisms, including plants and bacteria, and is widely distributed in both Gram-positive and Gram-negative bacteria^[^^1^^]^. Levansucrase from Gram-positive bacteria is secreted as a specific extracellular enzyme by two-step mechanisms as the enzyme possesses signal peptides cleaved after the carboxyl-terminal alanine during its export from the cytoplasmic to the outside of the cell^[^^2^^-^^4^^]^. In contrast, levansucrase from Gram-negative bacteria is fully secreted into its medium by a signal peptide-independent pathway involving the formation of an intermediate N-terminal protected amino acid in the periplasmic loop^[^^5^^,^^6^^]^. Levansucrase (EC 2.4.1.10) cleaves the β-1,2-glycosidic bond of sucrose and transfers the resulted fructosyl unit to numerous acceptor molecules, such as the short-chain acyl alcohols or mono- and di-saccharides^[^^7^^]^, as well as catalyzes the conversion of sucrose to fructan polymer. Levansucrase structure is supposed to be cell-wall anchoring motifs in their C-terminal domains, which are involved in the proteolysis^[^^8^^,^^9^^]^. 

Levan is one of the fructose polymers (fructan) linked by the β-2,6-fructofuranosidic bond, synthesized in transfructosylation reaction, and catalyzed by levansucrase using sucrose as a substrate^[^^10^^,^^11^^]^. Fructan, as a product of fructosyltransferase activity, can be obtained from various sources with different chemical compositions, molecular weights, and structures^[^^12^^,^^13^^]^. Levan is naturally synthesized by several flowering plant species, some bacteria, and fungi^[^^14^^,^^15^^]^. Due to the unique characteristics, levans are being used in various industries, including food, cosmetic, and medicinal products. Levan is nontoxic and highly soluble in water and possesses low viscosity, strong adhesivity, and film-forming ability, as well as compatiblility with salts and surfactants, heatstability, acid and alkali adaptability, biodegradablility, and biocompatiblility to other substances^[^^16^^-^^18^^]^. In comparison to the levan product in plants, those synthesized by bacteria often have a higher degree of polymerization^[^^19^^]^. Bacterial levans contains around 10^3^-10^4^ fructosyl units, whereas plant levans consist of about 10-200 fructosyl units^[^^20^^]^.

As the microbial levansucrase generates more branched levans, the levan products have larger molecular weight (~2-100 × 10^6^ Da) and more extensively branched when compared to levans from plants (2000-3300 Da)^[^^16^^,^^21^^,^^22^^]^. Furthermore, plants make their levans in vacuoles, while bacteria produce them extracellularly^[^^23^^]^. Levan, commonly found as a microbial exopolysaccharide, is produced by hydrolysis and transfructosylating activities of levansucrase^[^^24^^]^. Production of microbial levansucrase is greatly affected by fermentation parameters, such as sucrose concentration, nutrients sources, pH, temperature, time of cultivation, agitation, and aerations rates^[^^19^^,^^25^^,^^26^^]^. Currently, optimization of levan productions is a very interesting field of research and is important in developing an efficient and cost-effective biopolymer production process^[^^24^^]^. Since levan is a non-toxic polymer and can easily be synthesized* in vitro*, many researchers have focused on levan production using bacterial native extracellular levansucrase^[^^27^^]^. In addition, the data on levansucrase gene and protein structures are available^[^^19^^]^. However, levan production using recombinant levansucrase is rarely been reported. 

The wild types *Bacillus licheniformis* BK1 and BK2 are Gram-positive halophilic bacteria, which were isolated from Bledug Kuwu mud crater, Central Java, Indonesia. In our previous study, we successfully constructed the expression vectors pET-*lsbl-bk1* and pET-*lsbl-bk2* for high-level expression of levansucrase production^[^^28^^]^. In the present study, we aimed to investigate and optimize levan production catalyzed by recombinant levansucrase from *B. licheniformis* BK1 and BK2 expressed by pET-*lsbl-bk1* and pET-*lsbl-bk2* constructs in *Escherichia coli* host. Herein, we report the optimum condition of levan production by two recombinant enzymes. The potential uses of levan, as an antioxidant and antibacterial agent, were also studied.

## MATERIALS AND METHODS

**Strains, plasmids, and culture condition**

*E. coli* BL21 (DE3) pLysS (our collection), as the expression host and plasmids pET-30a(+) (Invitrogen, USA), pET-*lsbl-bk1* (GenBank: MF774877.1), and pET-*lsbl-bk2* (GenBank: MF774878.1) as expression vectors were used in recombinant production of target enzyme *Staphylococcus aureus*, *E. coli*, and *Pseudomonas aeruginosa* (obtained from Microbiology Study Program, Universitas Hasanuddin, Indonesia) were used in antibacterial assays. The *E. coli* strains were grown in LB medium containing 1% (w/v) tryptone (Liofilchem, Italy), 0.5% (w/v) yeast extract (Liofilchem), and 1% (w/v) NaCl (Merck, Germany). Kanamycin (Bio Basic, USA) was u for selecting recombinant *E. coli* cells. Chemicals employed in this study were IPTG (Thermo Fisher Scientific, USA), 3,5-dinitrosalicyclic acid (Sigma Aldrich, USA), sucrose, Na_2_HPO_4_, NaH_2_PO_4_.H_2_O, ethanol, phenol, H_2_SO_4_ 97%, DPPH, and ascorbic acid (Merck).

**High level** ***in vitro*** **synthesis of levan**

The in* vitro *levan synthesis was developed in two-stage processes. The first process was the heterologous overexpression of levansucrase gene cloned (pET-*lsbl-bk1* and pET-*lsbl-bk2*) in *E. coli* BL21 (DE3) pLysS host cell to produce levansucrase, which its conditions had been optimized in our previous research^[^^29^^]^. The second process was the catalytic reaction in a sucrose medium employing the produced levansucrase to enhance levan production. The optimizations of *in vitro *levan were performed using the RSM approach^[^^29^^,^^30^^]^.

**Heterologous expression of** ***B. licheniformis*** **levansucrase**

A single colony of *E. coli* BL21 (DE3) pLysS cells carrying pET-*lsbl-bk1* was inoculated into a 5-mL LB medium containing 50 μg/mL of kanamycin and 0.6% (w/v) NaCl. The culture was grown at 37 °C overnight. Then 2 mL of this overnight culture was inoculated into 100 mL of a fresh LB medium containing kanamycin and NaCl, incubated at 37 °C with aeration until the OD_600_ reached 0.6-1. The expression of levansucrase was induced by IPTG (0.6-mM), and the culture was further incubated at 42 °C for 4 hours. Different conditions were performed for pET-*lsbl-bk2*, including 1.1% (w/v) NaCl, 0.7 mM of IPTG, and 40 °C incubation temperature. The expressed enzyme is known to be secreted into the medium^[^^28^^]^. Therefore, the supernatant of the culture, i.e. the levansucrase crude extract, was collected by centrifugation at 6,082 ×g at 4 °C for 15 min. The obtained crude extracts were used in experiments to optimize the *in vitro *levan production by RSM. 

**Experimental design**

The RSM was applied to search for the optimum condition of levan production catalyzed by levansucrase Lsbl-bk2^[^^29^^]^. BW design was also used to optimize the *in vitro *levan production catalyzed by Lsbl-bk1. BW design and CCD are experimentally efficient technique for our study to develop a predictive model using experimental variables on *in vitro* levan production. In this design, the following three parameters were applied: the temperature of incubation (*X*_1_) between 20 °C to 54 °C, pH (*X*_2_) from 4 to 10, and sucrose concentration (*X*_3_) from 5% to 20% (w/v), as presented in Tables 1 and 2. The design was made up of BW full 2^3^ factorial with eight cube points, augmented with five center points and six-star points. Accordingly, the total runs included 20 experiments. Levan concentration (*Y*) was considered as a dependent (response) variable. To estimate the optimal point and generate the contour plots, a second-order polynomial function was fitted to the experimental results. The predicted values of the model were validated by experiments performed in five replicates and compared with the model predicted values. The Minitab statistical software (version 18) was used for the experimental designs and regression analysis of the experimental data.

***In vitro*** **levan production and determination of levan** **concentration**

The *in vitro *levan synthesis was conducted with various conditions (Table 1) by mixing 8 mL of levansucrase fresh crude extract with 8 mL of phosphate buffer containing sucrose substrate, filtered through a 0.45-μm filter before loading with enzyme crude extract. The reaction mixtures were incubated at the desired temperature for 24 hours, stopped by heating at 80-100 °C for 5 min, and cooled down to room temperature. The suspensions were then centrifuged at 6,082 ×g at 25 °C for 10 min^[^^30^^]^. The concentration of the produced levan in the supernatants was determined by colorimetric phenolic-sulfuric method at 490 nm using fructose as a standard via determining the amount of sugar produced from sucrose hydrolysis by levansucrase^[^^31^^,^^32^^]^. Briefly, 450 µL of levan solution was transferred into a tube, and 50 µL of 5% (w/v) phenol solution was added. The mixture was then vortexed to homogenize. In the next step, 500 µL of concentrated sulfuric acid was added rapidly and carefully. The tube was allowed to stand on ice bath for 5 min shaken, and placed in a 90 °C water bath for 5 min to stop the reaction. The mixture was cooled down to room temperature before reading the absorbance of the final yellow-orange color product at 490 nm. 

**Levan extraction**

The levan was extracted from the supernatant through the precipitation by adding three volumes of cold ethanol (3:1 v/v) at 4 °C overnight. The precipitate was recovered by centrifugation at 6,082 ×g at 4 °C for 20 min. To remove impurities, the precipitate was washed twice with ethanol 96% and once with deionized water, then freeze-dried for about 2 to 4 hours. The obtained levan was used for further analysis, such as morphological characterization, structure identification, thermal stability determination, and bioactivity.

**Levan analysis**

***Characterization of levan***

The morphology of the solid synthesized levan was analyzed by SEM (JEOL JSM 6510 LA, Japan), and its structure was characterized using FTIR (Shimadzu IR Prestige-21, Japan) and NMR spectroscopy (NMR spectrometer, Agilent 500 MHZ, USA) to obtain ^1^H NMR and ^13^C-NMR spectra. Besides, the characterization of levan thermal stability was determined under a nitrogen atmosphere using thermal gravimetric analysis (TG/DTG, Hitachi STA7300, USA). 

***Antioxidant activity of levan***

Antioxidant activity of levan was tested by DPPH free radicals scavenging assays^[^^33^^,^^34^^]^ using ascorbic acid as a standard. A fresh solution of DPPH was prepared by dissolving solid DPPH in ethanol absolute to obtain 0.5 mM of DPPH concentration. Ascorbic acid solutions were prepared in various concentrations (30, 120, 240, 420, 600, 960, and 1200 μg/mL), and levan solutions were prepared at the concentrations of 30, 250, 500, 1250, 2500, 3500, 4500, and 5000 μg/mL. Afterward, 250 μL of DPPH solution (0.5 mM) was added to 1.0 mL of the tested solution, which was then shaken vigorously to become homogeneous and incubated in the dark at room temperature for 30 minutes. The absorbance was read at 514 nm. Ethanol was utilized as a control solution. The percentage of inhibition was calculated using the following formula:

$\mathrm{\% inhibition}=\frac{\mathrm{The absorbance of the control}-\mathrm{Absorbance of the sample}}{\mathrm{The absorbance of the control}}\times 100$

(Eq. 1)

**Table 1:** The CCD experimental design for *in vitro *levan production catalyzed by Lsbl-bk1

**Parameters**	**Levels**				
	**-1,682**	**-1**	**0**	**+1**	**+1,682**
Temperature (°C)	20.0	27.5	37.0	47.4	54.0
Sucrose concentration (% w/v)	5.0	8.0	12.5	16.9	20.0
pH	4.0	5.2	7.0	8.7	10.0

The concentration of the sample with IC_50_ was calculated from the graph of % inhibition vs. sample concentration. 

***Antibacterial activity of levan***

The antibacterial activity of levan was assessed against three species, namely *E. coli*, *S. aureus*, and *P. aeruginosa*. The test was carried out using agar well diffusion method utilizing a metal cup with a diameter of 6 mm and height of 10 mm. In this experiment, 0.5% (b/v) NA supplemented with 0.3% (b/v) beef extract and 0.5% (b/v) peptone was used. Also, 1 mL of the fresh liquid culture containing about 10^5^-10^6^ CFU was spread onto the NA solid medium, where the well was made using a metal cup, which was then filled with 0.2 mL levan solution (10% and 20% w/v). The system was incubated at 37 °C for 24 h, and the antimicrobial activity was detected by measuring the inhibitory clear zones around the well^[^^35^^,^^36^^]^.

## RESULTS

**Optimization of high level in vitro levan synthesis**

The *in vitro* synthesis of levan was carried out by levansucrase utilizing sucrose as a substrate. The conditions of *in vitro *levan production were optimized to achieve high efficiency and high yield of levan through RSM. Three parameters, the temperature of incubation (*X*_1_), pH of the medium (*X*_2_), and sucrose concentration (*X*_3_), influencing the *in vitro *levan production catalyzed by Lsbl-bk1 were treated as independent variables. The values of the obtained response under different experimental conditions of BW 2^3^ factorial CCD are listed in Table 2. Optimization of *in vitro *levan production catalyzed by Lsbl-bk2 was not described as it had been reported in our previous study^[^^29^^]^. The results of CCD were fitted into second-order polynomial equation (equation 2) for the prediction of response on the basis of coded value.

$Y=-326.1+\mathrm{8.665}\left(x_{1}\right)+58.47\left(x_{2}\right)+10.43\left(x_{3}\right)-0.12{x_{1}}^{2}-4.19{x_{2}}^{2}-0.47{{(x}_{32206})}^{2}0.04\left(x_{1}x_{2}\right)+0.01\left(x_{1}x_{3}\right)+0.06\left(x_{2}x_{3}\right)$

(Eq. 2) 

Equation 2 reveals the relationship between levan concentration, as a dependent variable, obtained from linear or quadratic of the three independent variables. Model significance and role of each variable were evaluated using ANOVA, and the results were presented in Table 3. The mathematical model obtained (Equation 2) resulting *p* < 0.05, indicated statistically significant of this model. The ANOVA of the regression model gave both *R*^2^ and adjusted *R*^2^ values close to 1.00, further confirming that the model was highly significant to predict the response. Besides, the quadratic model was statistically significant for the description of levan yield where the *p *value is 0.000, which is less than 0.05. It can be seen from Table 3 that the linear and quadratic terms of regression coefficients on sucrose concentration (X_3_) were significantly influenced by the *in vitro *levan production because the probability value is smaller than 0.05. 

**Table 2:** BW 2^3^ factorial experimental results of *in vitro *levan production

**Run**	**Temp** **(°C)**	**pH**	**Sucrose concentration** **(% w/v)**	**Observed levan concentration** **(mg/mL)**
1	54.0	7.0	12.5	58
2	37.0	7.0	12.5	96
3	37.0	4.0	12.5	63
4	47.1	8.8	17.0	61
5	37.0	7.0	12.5	96
6	37.0	7.0	5.0	76
7	26.9	5.2	17.0	61
8	37.0	7.0	12.5	97
9	47.1	5.2	8.0	64
10	26.9	5.2	8.0	64
11	26.9	8.8	8.0	64
12	37.0	10.0	12.5	51
13	47.1	8.8	8.0	61
14	37.0	7.0	12.5	96
15	20.0	7.0	12.5	64
16	47.1	5.2	17.0	63
17	37.0	7.0	20.0	60
18	37.0	7.0	12.5	96
19	26.9	8.8	17.0	63
20	37.0	7.0	12.5	96

Temp, temperature

**Table 3:** Analysis of variance for *in vitro *levan production catalyzed by Lsbl-bk1 (*Y*) as the function of temperature (*X*_1_), pH (*X*_2_), and sucrose concentration (*X*_3_)

**Source**	**Sum of squares**	**Degrees of freedom**	**Mean square**	**F-value**	***p*** ** value**
Model	5018.78	9	557.64	51.42	0.000
Linear	121.89	3	40.63	3.75	0.049
* X*_1_	9.33	1	9.33	0.86	0.375
* X*_2_	34.14	1	34.14	3.15	0.106
* X*_3_	78.43	1	78.43	7.23	0.023
Square	4889.84	3	1629.95	150.31	0.000
* X*_1_ *X*_1_	2017.58	1	2017.58	186.06	0.000
* X*_2_ *X*_2_	2558.31	1	2558.31	235.92	0.000
* X*_3_ *X*_3_	1245.50	1	1245.50	114.86	0.000
2-way interaction	7.05	3	2.35	0.22	0.883
*X*_1_*X*_2_	4.16	1	4.16	0.38	0.550
*X*_1_*X*_3_	1.09	1	1.09	0.10	0.758
*X*_2_*X*_3_	1.80	1	1.80	0.17	0.692
Error	108.44	10	10.84		
Lack-of-fit	107.82	5	21.56	174.01	0.000
Pure error	0.62	5	0.12		
Total	5127.22	19			

*R* ^2^ = 0.9789; *R*^2 ^(adjusted) = 0.9598

**Fig. 1:**
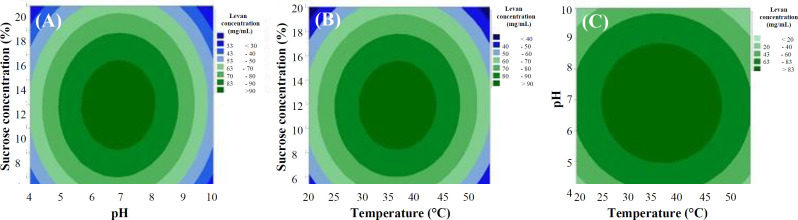
Contour plots predicting *in vitro *levan production as the function of pH and sucrose concentration (A), temperature and sucrose concentration (B), and temperature and pH (C).

The three-dimensional response surface described by the contour plots model is presented in Figure 1. The highest levan concentration is predicted to be at 36 ^o^C, 96.268 mg/mL levan. Five experiment replicates were then performed under this predicted optimum condition, and the obtained levan concentration was 96.117 mg/mL on average. 

**Analysis of levan structure**

**Fig. 2.**
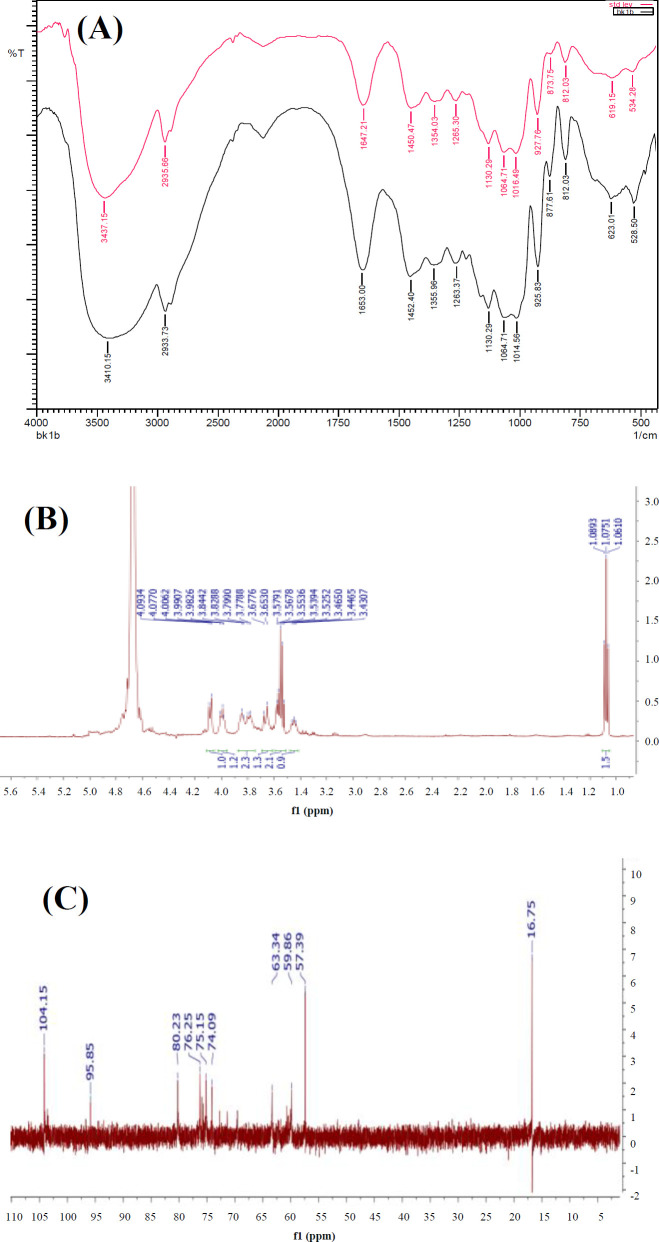
Analysis of the produced levan catalyzed by Lsbl-bk1: (A) FTIR spectrum of levan (black line) compared with levan standard from *E*. *herbicola* (red line), (B) levan ^1^H-NMR spectrum, and (C) levan ^13^C-NMR spectrum

The structure of levan was verified by FTIR and NMR, as presented in Figure 2. In FTIR, absorption characteristic of levan (Fig. 2A) was attributed by the O-H stretching at around 3447-3414 cm^-1^, C-H stretching at around 2931.80 cm-1, and C=O stretching at 1658.78 cm-1. The fingerprint pH 7, and 12% (w/v) of sucrose, which produced spectrum between 1271.01 cm^-1^ and 925.83 cm^-1^ corresponded to carbohydrate molecules and related to overlapping signals of glycosidic (C-O-C) stretching. The levan spectra, ^1^H-NMR, and ^13^C-NMR can be seen in Figures 2B and 2C. The levan ^1^H-NMR spectrum exhibited the signal characteristic between 3.4 and 4.3 ppm, associated with sugar protons (Fig. 2B). The six broad resonance signals of levan ^13^C-NMR spectrum at 104.1 (C2), 80.2 (C5), 76.2 (C3), 75.1 (C4), 63.3 (C6), and 59.8 (C1) ppm corresponded to the carbon of β-fructofuranose, confirming that levan has successfully been synthesized.

**Morphology analysis**

**Fig. 3:**
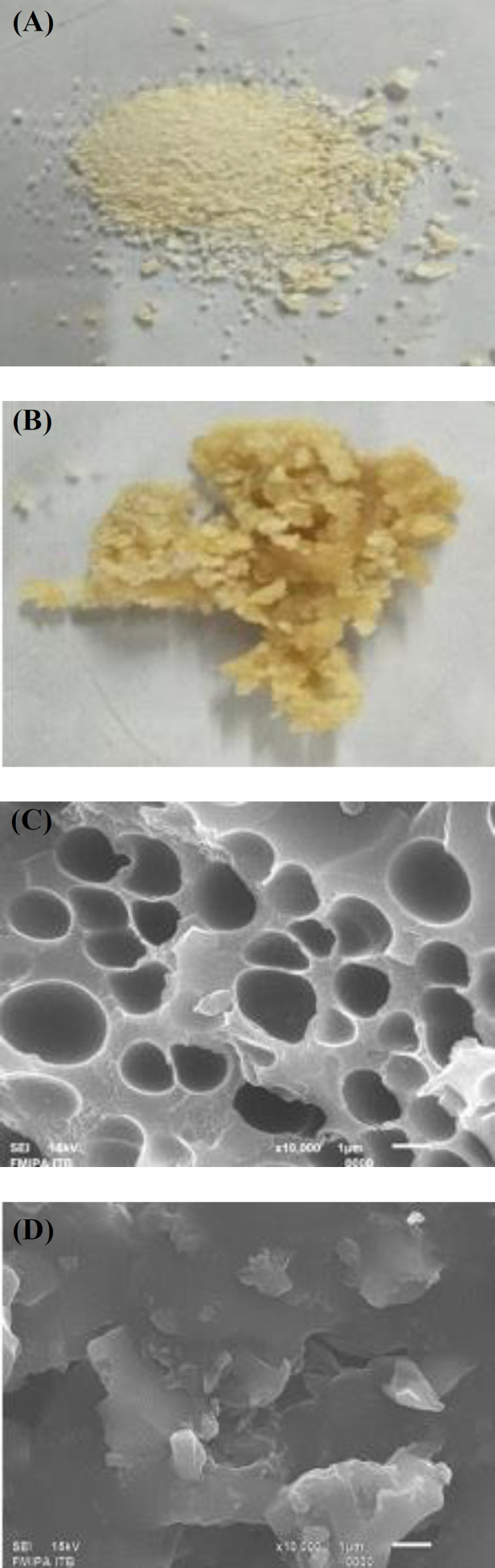
The morphological characteristic of solid produced levan catalyzed by (A) Lsbl-bk1 and (B) Lsbl-bk2 and the SEM image of levan catalyzed by (C) Lsbl-bk1 and (D) Lsbl-bk2

The *in vitro* levan product catalyzed by Lsbl-bk1 and Lsbl-bk2 were extracted with ethanol. The resulted levan showed different solid appearances, and then SEM was examined. The product of Lsbl-bk1 appeared as a white granule (Fig. 3A) with irregular circular pores, while the Lsbl-bk2 product showed yellow solid and a rather sticky texture (Fig. 3B), with a sheet-shaped solid without any pores. These morphology differences might be due to different synthesis conditions, particularly the pH, which would influence the morphological characteristic of the synthesis polymer^[^^29^^]^. 

**TG and DTG analysis**

**Fig. 4:**
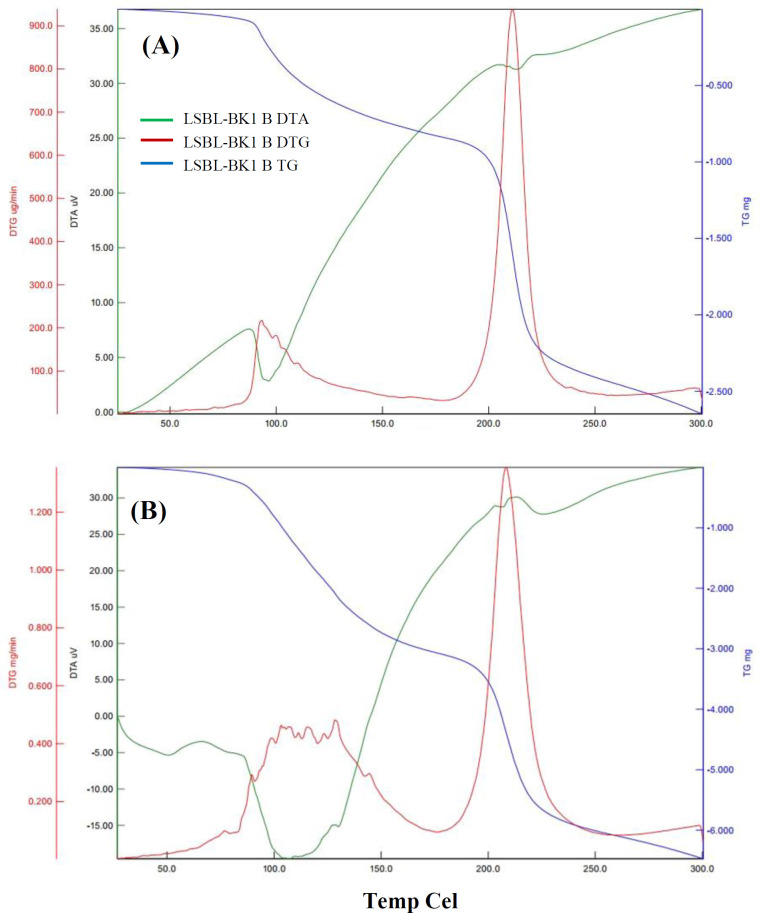
TG and DTG curves of the produced levan catalyzed by (A) Lsbl-bk1 and (B) Lsbl-bk2. Blue, red, and green lines show TG, DTG, and DTA curves, respectively

TG analysis and DTG profiles were used to investigate the thermal stability of the produced levan. The obtained thermograms of the *in vitro* produced levan catalyzed by Lsbl-bk1 and Lsbl-bk2 are presented in Figure 4, showing three stages of levan mass reduction at 0-100 °C, 100-200 °C, and 200-300 °C. The first region, 0-100 °C, is attributed to water and ethanol evaporation. Both levans attained their weight in the second region, 100-200 °C, indicating its thermal stability. The third region, 200-300 °C, which showed around 50% of mass reduction, might be corresponded to levan degradation. The DTG curves demonstrate that the main peak of levan degradation occurred at 211 °C and 208 °C for levan of Lsbl-bk1 and Lsbl-bk2, respectively. 

**Antioxidant activity**

**Fig. 5:**
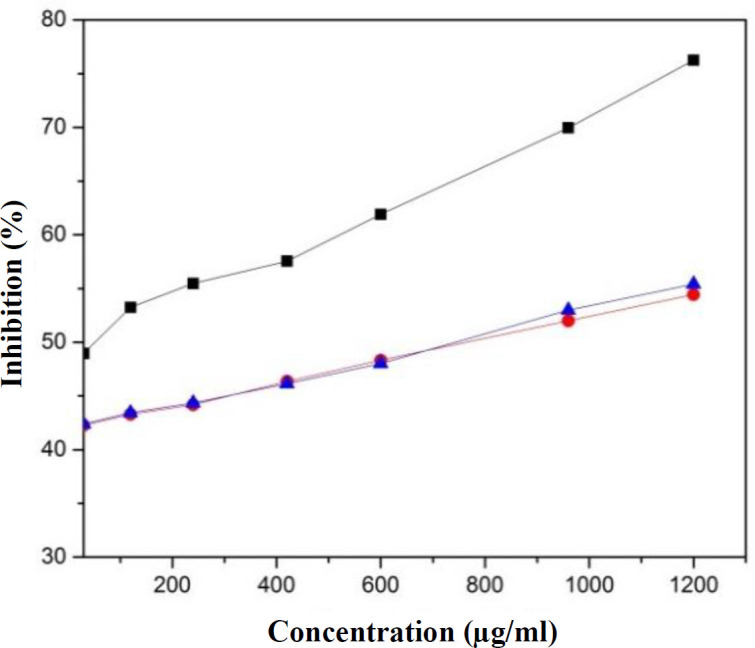
Antioxidant activities of ascorbic acid (black rectangle), levan Lsbl-bk1 (red circle) and levan Lsbl-bk2 (blue triangle)

The antioxidant activity of the produced levan catalyzed by Lsbl-bk1 and Lsbl-bk2 was evaluated by DPPH free radical scavenging method. The results exhibited that the percentage activity of both levans is at the concentration of 30-1200 μg/mL, which could inhibit around 55% DPPH activity compared to ascorbic acid, which effectively inhibited up to 76% at the same concentration (Fig. 5). The scavenging activities of both levans directly increased to 75% if the concentration increased up to 5 mg/mL. 

**Antibacterial activity**

**Fig. 6:**
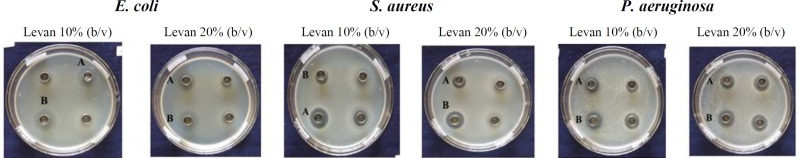
Growth inhibitions of *E. coli*, *S. aureus*, and *P. aeruginosa* in levan containing nutrient agar. A and B show produced levan catalyzed by Lsbl-bk1 and Lsbl-bk2, respectively

Antibacterial activity of the levan products was studied against *E. coli*, *S. aureus*, and *P. aeruginosa*. The results are depicted in Figure 6. The antibacterial activities were observed as a clear zone around the cup filled with levan. It can be seen in the Figure that both levans possessed antibacterial activity. Levan produced by Lsbl-bk1 catalysis showed the highest activity against *S. aureus* (12-16 mm), whereas those catalyzed by Lsbl-bk2 had the highest activity against *E. coli *(15-16 mm) and *P. aeruginosa *(14-16 mm). The appearance of the inhibition zone was not broad and not strong enough to necessarily kill the bacteria, indicating that levan owes a bacteriostatic effect on these tested bacteria. 

## DISCUSSION

BW design has been proved to be an efficient technique for optimization of the *in vitro* levan production catalyzed by levansucrase Lsbl-bk1, using three variables (temperature, pH, and sucrose concentration). The interaction between the three tested independent variables revealed no significant effect on *in vitro *levan production, as indicated by the *p*>0.05 (Table 3). The same result was also achieved from the *in vitro* levan production catalyzed by levansucrase Lsbl-bk2^[^^29^^]^. It is of advantageous that the three studied variables are non-interacting one to the other; hence, the changes of one variable would minimally affect the others.

Our study indicated that the optimum condition for the *in vitro *levan synthesis catalyzed by Lsbl-bk1 was at 36^o^C, pH 7, and 12% (w/v) sucrose, while this condition for Lsbl-bk2 was at 32^o^C, pH 8, and 12% (w/v) sucrose^[^^29^^]^. However, the *in vitro* levan synthesis by both levansucrases gave a similar high yield, which was about 95 mg/mL. Lu *et al.*^[^^30^^]^ reported that the optimum condition for *in vitro *levan production catalyzed by recombinant levansucrase from *B. licheniformis* 8-37-0-1 occurred at 0.8 M sucrose concentration (equivalent to 14% w/v) in pH 6.5 at 40 °C for 24 hours with a yield of 7.1 mg/mL levan. These dissimilarities might be due to the different environmental origins of *B. licheniformis* 8-37-0-1, which was isolated from the hydrothermal vent of Panarea Island (Italy)^[^^37^^]^, but our strain was obtained from Bledug Kuwu mud crater, Central Java, Indonesia. The *in vitro *production of levan in our research appeared to be more efficient, which resulted in higher levan yield at lower temperatures and lfor of *in vitro *levan production is more applicable and more feasible to industries.

The FTIR spectrum of levan catalyzed by Lsbl-bk1 was similar to that of levan from *Erwinia herbicola*^[^^38^^] ^and consistent with the spectrum of levan obtained by the catalysis of Lsbl-bk2^[29]^. ^1^H-NMR and ^13^C-NMR spectra showed similar signals with the levan produced by *Acetobacter xylinum* NCIM2526 and *Brenneria goodwinii*^[^^39^^,^^40^^]^. The obtained FTIR and NMR patterns indicated that levan produced by the recombinant of *B. licheniformis* levansucrase represented β-(2,6)-levan polysaccharide.

The morphological properties of both synthetic levans were different due to variations in the synthesis of optimum conditions, especially pH. Accordingly, the synthesis of pH-responsive biopolymer was beneficial to develop preferred materials for drug delivery or other industrial desired materials^[^^41^^]^. Moreover, the thermal stability properties of levan produced were degraded at lower temperatures (208 ^o^C and 211 ^o^C) compared to other levans, such as levan produced by *Zymomonas mobilis* and *Halomonas* sp. AAD6 that degraded at 217^o^C and 253 °C, respectively^[^^42^^,^^43^^]^. 

The bioactivity of both levans has been tested in our study as antimicrobial and antioxidant agents. The antioxidant capacity of our levan was similar to that of Srikanth *et al.*’s^[^^16^^] ^and Benattouche *et al.*’s^[^^44^^]^ studies, in which the DPPH-scavenging effect increased with the elevation of levan concentration as exopoly-saccharides. The scavenging ability of levan on DPPH radical might be due to the ability of hydroxyl group to donate its electron as hydrogen radical^[^^27^^,^^34^^,^^45^^]^. These results suggest that the antioxidant activity of both levans is relatively lower compared to ascorbic acid. However, it would be possible and might be beneficial to be used in biomedicine industries. Aside from that, both Lsbl-bk1 and Lsbl-bk2 levan products, as exoploysaccharides, have beneficial potency as an antibacterial agent. Previous studies have shown that exopolysachharides with antimicrobial activity could inhibit the growth of *S. aureus*, *E. coli, *and *P. aeruginosa *with an inhibition zone diameter about 15-20 mm^[^^44^^,^^46^^]^. It has also been assumed that exopolysaccharide levans can disrupt the bacterial cell wall integrity by blocking nutrients input^[^^36^^,^^46^^]^.

In summary, our preliminary research indicates that the produced levan possessed antioxidant and antibacterial activities; therefore, it is the potential to be used in food and pharmaceutical industries.
